# Severe Congenital Factor X Deficiency as a First Case Report in Cambodia

**DOI:** 10.1155/crh/5592395

**Published:** 2025-08-21

**Authors:** Chin Soey, Meang Sovandos, Lam Pechkethia, Lean Kimsreng, Chean Sophâl

**Affiliations:** ^1^Department of Pediatric Hematology and Immunology, National Pediatric Hospital, Phnom Penh, Cambodia; ^2^Technical Office, National Pediatric Hospital, Phnom Penh, Cambodia

**Keywords:** bleeding disorder, Cambodian, child, Factor X deficiency

## Abstract

**Background:** Factor X (FX) deficiency is a rare autosomal recessive inherited bleeding disorder, with an estimated prevalence of approximately 1 in 1,000,000 individuals. According to the most recent data published by the World Federation of Hemophilia, no cases of FX deficiency have been reported in Cambodia to date.

**Case Report:** A 14-year- and 7-month-old Cambodian boy presented with recurrent gum bleeding. His medical history was notable for multiple hematomas, joint ankylosis, and blue sclera. He was born to second-degree consanguineous parents, with no known family history of bleeding disorders. Laboratory evaluation revealed prolonged prothrombin time (PT) and activated partial thromboplastin time (APTT) and severely reduced FX activity (< 1%), consistent with a diagnosis of severe congenital FX deficiency. Bleeding was successfully managed with fresh frozen plasma, initially administered at 15 mL/kg, followed by maintenance doses of 5 mL/kg twice daily.

**Conclusion:** FX deficiency, though rare, should be considered in the differential diagnosis of pediatric patients presenting with recurrent gingival or mucocutaneous bleeding in conjunction with prolonged PT and APTT. This consideration is particularly important in resource-limited settings such as Cambodia, especially in children born to consanguineous parents and after more common coagulopathies have been excluded. In low-resource settings, where FX concentrates are often unavailable or unaffordable, fresh frozen plasma remains the primary treatment option.

## 1. Introduction

Factor X (FX) deficiency is an infrequent inherited bleeding disorder [1]. It is an autosomal recessive condition, with an estimated prevalence of 1 in 1,000,000 individuals in the general population [2]. FX deficiency arises due to insufficient production of the FX protein. Clinically, it is stratified into severe, moderate, and mild forms depending on activity levels measured by functional assays: severe (< 1%), moderate (1%–4%), and mild (6%–10%) [1]. The severity of the disease is directly correlated with residual protein activity. For effective hemostasis, an FX level > 10% is generally considered sufficient [3].

The most common bleeding symptoms associated with FX deficiency are mucocutaneous, including easy bruising, epistaxis, gum bleeding, menorrhagia, hematuria, hematomas, hemarthrosis, gastrointestinal bleeding, intracranial hemorrhage, and umbilical cord bleeding [4]. Individuals with mild FX deficiency are often asymptomatic and may be diagnosed only during routine screenings or due to a positive family history. Those with moderate deficiency may be identified following hemostatic challenges such as surgery, trauma, or menstruation. Symptoms can vary in severe cases but typically manifest at or shortly after birth [5].

As with other rare disorders, FX deficiency is often unrecognized or misdiagnosed [5]. According to the latest published census from the World Federation of Hemophilia (WFH), there have been no reported cases of FX deficiency in Cambodia [6]. This article presents the first documented case of FX deficiency in a Cambodian child.

## 2. Case Report

A 14-year- and 7-month-old boy of Cambodian origin, with second consanguineous parents, was referred to our center due to ongoing gum bleeding. As a single child in the family, he was born at term via normal delivery to a healthy mother. He is developing and growing normally, but he experienced multiple subcutaneous hematomas, including a hematoma of the right thigh muscle and leg, associated with frequent gum bleeding episodes and subsequently ankylosis of the wrist and knee since turning 4, and there has been no family history of bleeding disorders.

The physical examination revealed blue sclera and ongoing gum bleeding with hematoma, while cardiovascular and pulmonary examinations were unremarkable, with clear sounds and a regular rhythm. The gastrointestinal examination showed no signs of hemorrhagic digestive manifestations or organomegaly.

CBC showed microcytic anemia (Hb 7.2 g/dL, MCV 68 fL) with normal platelet count. Coagulation screening revealed prolonged PT and APTT; with FX activity < 1% confirming severe FX deficiency (details in Table 1). Liver enzyme levels were within normal limits, with AST at 25 U/L and ALT at 9 U/L. Initial coagulation screening showed a prolonged prothrombin time (PT) of 28.2 s and an activated partial thromboplastin time (APTT) of 44.9 s. Given the suspicion of endogenous vitamin K deficiency—potentially affecting Factors II, VII, IX, and X—a single 5 mg dose of vitamin K1 was administered. However, the patient continued to experience persistent gum bleeding, indicating a lack of clinical response. As a result, extended coagulation studies were performed, focusing on Factors I, II, V, VII, and X, all of which can contribute to concurrent prolongation of PT and APTT. These studies revealed a markedly reduced FX activity of less than 1% (undetectable), with no detectable FX inhibitor, confirming a diagnosis of severe congenital FX deficiency. The other results of blood work are summarized in Table 1. Molecular genetic testing was not performed on the patient or his parents due to the unavailability of the tests in Cambodia. Treatment with fresh frozen plasma (FFP), initiated at 15 mL/kg followed by maintenance doses of 5 mL/kg twice daily, effectively controlled the bleeding. The patient showed gradual resolution of gum hematomas and was subsequently discharged. He has since been monitored through multiple follow-up visits.

## 3. Discussion

FX, also known as Stuart–Prower factor, is a vitamin K-dependent coagulation factor synthesized in the liver that requires post-translational modification for maturation [7]. The active form, FXa, acts as a catalytic serine protease and is the primary activator of prothrombin [8]. It is located at the junction of the extrinsic and intrinsic pathways. FX is central to the coagulation cascade, and its deficiency prolongs both PT and APTT, as seen in our case [9].

A diagnosis of FX deficiency is generally based on identifying characteristic symptoms, a detailed history of the patient and family, thorough clinical evaluation, and confirmed by any of the following specialized tests: functional activity assays, immunological assays, or chromogenic assays [1], whereas the molecular genetic testing is typically unnecessary [5]. FX deficiency, though uncommon, should be included in the differential diagnosis in similar clinical scenarios, particularly in resource-limited settings like Cambodia. The risk is elevated in populations with a higher prevalence of consanguinity, as observed in our patient's family history. A FX functional activity assay, which is readily available in our setting, can be a practical diagnostic tool, as demonstrated in our patient's case.

Individuals with severe FX deficiency may experience recurrent gum bleeding and soft tissue hemorrhages involving muscles and joints, reported in approximately 28% of cases. If diagnosis is delayed, these bleeding episodes can lead to long-term complications such as joint ankylosis and muscle fibrosis or scarring [10, 11], as observed in our patient. Additionally, the presence of microcytic anemia (hemoglobin 7.2 g/dL, MCV 68 fL) is likely attributable to chronic blood loss secondary to unrecognized or recurrent mucocutaneous and internal bleeding.

The incidence of FX deficiency is estimated at 1 in 500,000 to 1 in 1,000,000 individuals worldwide, but it is significantly higher in populations with a high prevalence of consanguineous marriages, such as in the Middle East and parts of South Asia [12]. FX deficiency follows an autosomal recessive inheritance pattern [5], meaning the disorder occurs when an individual inherits two pathogenic variants of the F10 gene—one from each parent. Individuals with only one defective copy are typically asymptomatic carriers. In our case, both parents were likely heterozygous carriers of the F10 variant, conferring a 25% risk of having an affected child. However, the genetic status of the patient's parents remains unknown, as testing was not available in our setting. This highlights the need for genetic counseling, particularly in high-risk populations, to educate families about inheritance patterns of this disorder and the potential risks to future offspring. In women with FX deficiency, pregnancy poses a high risk of complications, including miscarriage, preterm labor, and hemorrhage [13].

FX deficiency is typically managed with replacement therapy using FFP, prothrombin complex concentrates (PCC), or plasma-derived FX (pdFX) concentrate [11]. The recommended initial dose of FFP is 10–20 mL/kg, followed by 3–6 mL/kg every 12 h to maintain trough FX levels above 10%, which is generally sufficient for hemostasis. While PCC and some Factor IX (FIX) concentrates contain therapeutic levels of FX, their efficacy varies. PCC with an FX:FIX ratio of 1:1 can increase plasma FX levels by approximately 1.5% per unit/kg. Single-factor replacement therapy is generally preferred for inherited bleeding disorders, as it provides targeted treatment with a lower risk of thromboembolic events [14]. However, in resource-limited settings like Cambodia, where highly purified FX concentrates are unavailable, FFP remains the primary treatment option.

## 4. Conclusion

FX deficiency, though rare, should be considered in the differential diagnosis of pediatric patients presenting with recurrent gingival or mucocutaneous bleeding in conjunction with prolonged PT and APTT. This consideration is particularly important in resource-limited settings such as Cambodia, especially in children born to consanguineous parents and after more common coagulopathies have been excluded. Genetic counseling is strongly recommended for affected families, particularly those planning future pregnancies. In low-resource settings, where FX concentrates are often unavailable or unaffordable, FFP remains the primary treatment option. According to similar case discussions in previously published literature [15], the diagnostic and management approach in this case aligns with recognized global standards.

## Figures and Tables

**Table 1:** Results of laboratory investigations.

Blood work	Results	Reference range
*Complete blood count*		
White blood cells	5.4	4–10 × 10^9^/L
Neutrophils	2.97	2–8 × 10^9^/L
Lymphocyte	1.72	1–5 × 10^9^/L
Red blood cells	3.52	4.5–5.5 × 10^12^/L
Hemoglobin	7.2 (↓)	13–17 g/dL
Hematocrit	21	40%–45%
Reticulocytes	1.2	0.5–1.5 × 10^9^/L
MCV	68 (↓)	83–100 fL
MCH	21	27–32 pg
Platelet count	401	150–410 × 10^9^/L
RDW-CV	17	11.5%–14%

*Liver enzymes*		
AST	25	< 31 U/L
ALT	9	< 32 U/L

*Renal functions*		
Urea	13	10–50 mg/dL
Creatinine	0.6	0.5–0.9 mg/dL

*Coagulation profiles*		
Bleeding time	3.5	2–7 min
PT	28.2 (↑)	10–16 s
APTT	44.9 (↑)	24–36 s
Factor I activity (fibrinogen)	4.4	2–4 g/L
Factor II activity	100	70%–120%
Factor V activity	80	70%–120%
Factor VII activity	81	70%–120%
Factor X activity	< 1 (↓)	70%–120%

*Note:* RDW-VC: red cell distribution width-coefficient of variation, ALT: alanine aminotransferase, AST: aspartate aminotransferase, (↑): increase, (↓): decrease.

Abbreviations: APTT, activated partial thromboplastin time; MCH, mean corpuscular hemoglobin; MCV, mean corpuscular volume; PT, prothrombin time.

## Data Availability

Patient health information supporting this case report is available upon request from the corresponding author, and all other relevant data are included within the manuscript.
